# COVID-19 vaccines: Current evidence and considerations

**DOI:** 10.1016/j.metop.2021.100124

**Published:** 2021-09-11

**Authors:** Alireza Tavilani, Ebrahim Abbasi, Farhad Kian Ara, Ali Darini, Zahra Asefy

**Affiliations:** a Islamic Azad University, Hamedan Branch, Hamedan, Iran; b Department of Clinical Biochemistry, Hamadan University of Medical Sciences, Hamadan, Iran; c Medical Biology Research Center, Kermanshah University of Medical Sciences, Kermanshah, Iran; d Department of Nanotechnology, Pharmaceutical Sciences Branch, Islamic Azad University (IAUPS), Tehran, Iran; e School of Nursing and Allied Medical Sciences, Maragheh University of Medical Sciences, Maragheh, Iran

**Keywords:** SARS-CoV-2 vaccine, 2019-nCoV vaccines, COVID-19 vaccines

## Abstract

The coronavirus disease 2019 (COVID-19) pandemic is a global crisis, with devastating health, business and social impacts. Vaccination is a safe, simple, and effective way of protecting a person against COVID-19. By the end of August 2021, only 24.6% of the world population has received two doses of a COVID-19 vaccine. Since the emergence of COVID-19, several COVID-19 vaccines have been developed and approved for emergency use. Current vaccines have shown efficacy with low risk of adverse effects. However, COVID-19 vaccines have been related to a relatively small number of cases of heart inflammation, anaphylaxis (allergic reactions), and blood clots formation. On the other hand, COVID-19 vaccination is not recommended for children less than 12 years of age. Furthermore, It has been proposed that some new variants (e.g., Lambda and Delta) are proficient in escaping from the antiviral immunity elicited by vaccination. Herein we present current considerations regarding the COVID-19 vaccines including: efficacy against new variants, challenges in distribution, disparities in availability, dosage gender and race difference, COVID-19 vaccine transport and storage, limitations in children and pregnant women. Long-time monitoring is essential in order to find vaccine efficacy and to rule out related side effects.

## 1 Introduction

Numerous medicines have been used for the treatment of coronavirus disease 2019 (COVID-19) during the past year. Although most of the medicines failed to show efficacy in treating COVID-19, researchers have encouraged herd immunity to control the current pandemic [1,2]. Vaccination is a safe, simple, and effective way of protecting a person against COVID-19. Although a massive number of experiments have been done since the virus was first recognized, there are still many unknowns about this COVID-19. Certain persons including pregnant women, breastfeeding individuals, autoimmune conditions and immunocompromised persons, diabetic patients, and people with respiratory and heart disease require special consideration for COVID-19 vaccination [[3], [4], [5], [6]]. Having certain medical conditions can make a person more likely to get severely ill from COVID-19 [2].

The effects of vaccines on the COVID-19 pandemic depend on various factors, including the efficiency; how rapidly they are manufactured, approved, and delivered; the immunity against new variants and how many subjects get vaccinated. Various health organizations are working to help confirm that approved COVID-19 vaccines are as effective as possible, so that they can have the most significant effect on the COVID-19 pandemic. A vaccine is a vital tool in the battle against COVID-19 infection, and there are many lifesaving and public health benefits to using the tools we now have [7,8].

At present, 184 candidate vaccines were being evaluated in preclinical and 104 in clinical stages of development. Furthermore, there are 41 vaccines in phase 3 and 18 COVID-19 vaccines approved, and are currently in use worldwide. These vaccines are in four primary groups using various platforms: (1) viral vector vaccines, (2) whole virus vaccines, (3) nucleic acid vaccines, and (4) protein-based vaccines [9]. Table 1 depicts the main characteristics of the currently available vaccines.

**Table 1:** Comparison of Pfizer/BioNTech, Moderna, Johnson & Johnson, and AstraZeneca vaccines [7,33].

Name of vaccines	Pfizer-BioNTech vaccine	Moderna	Johnson & Johnson	AstraZeneca
**Type of Vaccine**	mRNA vaccine	mRNA vaccine	Vector vaccine	Adenovirus vector vaccines
**Storage**	Stored for 6 months at −70 °C. Undiluted vials can be stored at room temperature for no more than 2 h.	Stored for 30 days between 2 °C and 8 °C.	Stored for up to 3 months between 2 °C and 8 °C.	Store in a refrigerator (2–8 °C). Do not freeze. Preserve the vials from light.
**Effectiveness**	95% in preventing the COVID-19 infection.	94.5% in preventing the COVID-19 infection.	85% in preventing the COVID-19 infection.	70% in preventing the COVID-19 infection.
**Number of Injections**	2 shots, given 21 days apart.	2 shots, given 28 days apart.	One dose is needed.	2 shots, given 28 days apart.
**Age Group**	People age 16 and older.	People age 18 and older.	People age 18 and older.	People age 18 and older.
**Variants**	Quite effective against the South African, UK variant, and Latin American variants.	Quite effective against the South African, UK variant, and Latin American variants.	Less effective against the South African and Latin American strains.	Less effective against South African variant, but appears effective against Brazilian and UK variants.
**Side effects**	➢ Swelling, pain, and redness at the site of vaccine. Fatigue, headache, fever, vomiting, redness, chills, lymphadenopathy, joint pain, paroxysmal ventricular arrhythmia, shoulder injury, lymphadenopathy, syncope, diarrhea, right axillary ➢ arrhythmia, and ➢ leg paresthesia.	Swelling, pain, and redness at the site of vaccine. Fatigue, headache, fever, vomiting, chills, myalgia, urticarial, and arthralgia. Bell's palsy and facial swelling has also been reported.	Swelling, pain, and redness at the site of vaccine. Nausea, vomiting, tiredness, muscle pain, chills, headache, and fever.	Swelling, pain, warmth, itching or bruising, and redness at the site of vaccine. Fatigue, headache, fever, vomiting, diarrhea nausea, chills, joint pain, muscle ache. Vaccine induced thrombotic thrombocytopenia, which are estimated to occur in 1 in 100,000 vaccinated people.

Although the striking amount of experiments carried out since the COVID-19 was first recognized, there are still a huge number of unknowns about this disease. Hence, there are multiple concerns about COVID-19 vaccines [8]. In the next section, we will discuss about vaccination in view of gender and race difference, new variants, efficacy and immunity, safety, dosage, transport and storage, distribution, vaccination in special groups, and virus transmission in vaccinated people.

## 2 Vaccination in view of gender difference

It has been shown that several factors, including the genetic, immune system, gut microbiome, and steroid hormones are varied between men and women that contribute to gender - and sex-specific vaccine responses and outcomes. Women produce more antibodies as a result of vaccination and respond more actively to infections. In women, a strong response of the immune system may increase the risk of autoimmune diseases and a good capability to fight against various infections. A higher level of COVID-19 antibody has been reported in women than in men after COVID-19 infection. Women display more strong cellular and humoral-mediated immune responses to vaccination and infection when compared to men [10]. Thus, the vaccine efficacy suggested for adults is potentially greater for women than men. Men, due to high levels of testosterone, show low levels of COVID-19 vaccine effectiveness. In this respect, males may need more doses of the COVID-19 vaccine compared with females [10].

## 3 Vaccination in view of race difference

Among those reported, the ethnic and racial distribution of the sample was not always stated, and methods are different, which may affect the results [11]. Asian, Hispanic, and Black people are infected with COVID-19 more than White ethnicity, with a possible relationship of higher risk of mortality and intensive care unit (ICU) admission in Asians [10]. Black females and males were about 4.2 times more likely to die from COVID-19 infection than White females and males [10]. However, in the UK, the mortality risks do not apply to Black ethnicity alone. Ethnicities of the people of Indian, Bangladeshi, Iranian, Pakistani, and Mixed had substantially increased risk of death by COVID-19 infection when compared with the White ethnicity [10].

## 4 COVID-19 vaccines and variants

RNA viruses such as the novel coronavirus are known for mutating and evolving quickly. RNA replication is more error-prone compared to DNA replication, so mutations happen commonly during copying. Sometimes the random mutation is beneficial for the virus, which helps it evade the host's immune system and infect new species or systems. A new variant of novel coronavirus emerged with a high number of mutations. The new variants are B.1.1.7 (Alpha), B.1.351 (Beta), P.1 (Gamma), B.1.617.2 (Delta), and C.37 (Lambda). The new variants are spread more easily, lead to severe disease, and may change the efficiency of COVID-19 vaccines [12].

These variants may be associated with a higher mortality rate. There is concern that the available COVID-19 vaccines may not provide sufficient immunity against new variants.

The vaccines are expected to protect subjects against new virus variants and effective at preventing severe respiratory disease and death. An update of vaccine composition may be necessary in order to maintain high efficacy against new variants. Furthermore, the revaccination schedule may also be essential if variants develop that are potentially different from the original coronavirus that the vaccines were produced against. Another variant, B.1.1.7, revealed in the UK, has been reported to have a high mortality rate and faster transmission speed. New variants reported in various countries can decrease the efficiency of the current COVID-19 vaccines. If the pandemic persists, the mutations of coronavirus will increase, and humanity must struggle for vaccination and worldwide distribution [13].

## 5 COVID-19 vaccines efficacy and immunity

No vaccine is 100% effective. There's no report so far that the COVID-19 vaccine can prevent transmission, but it can help protect against COVID-19 infection. Various countries have reported that the numbers of new cases and transmission rates of COVID-19 have reduced in many areas, probably due to the protective efficacy of vaccines and/or restrictions. However, the vaccine candidates have been evaluated in isolation, which makes it challenging to compare the efficiency of different vaccines. Therefore, it would be premature to hail the immunogenicity and safety observed in vaccine trials as a real achievement [14]. None of the approved COVID-19 vaccines contain the live virus that causes COVID-19. This means these vaccines cannot lead to COVID-19 infection. Generally a few weeks after vaccination, the body builds immunity against COVID-19 infection. Hence, it is possible for people to be infected with COVID-19 just before or after vaccination and yet get sick with COVID-19. This is because the COVID-19 vaccine has not yet had an adequate period to provide protection [15].

It has been reported that mRNA COVID-19 vaccines provide immunity for at least 6 months [16]. All COVID-19 vaccines have only been produced in the past months, It's too early to judge the duration of the immunity of these vaccines. Available findings [17,18] show that most patients who recover from disease develop an immune response against COVID-19 infection that provides about five to eight months of protection– although the exact immunity levels and protection period are not measured. Under normal conditions, phase 3 of vaccine studies could have continued for another few years, displaying how long protection lasts before the vaccine was distributed to the general community. The current COVID-19 vaccines are all two-dose vaccines (except for the vaccine from Johnson & Johnson). Appropriate immune response has been reported within about two weeks after the first dose. And the second dose then significantly increases the immune response and a shorter time after the second dose [15].

## 6 COVID-19 vaccines safety

The safety of the COVID-19 vaccine should be evaluated in participants of different ages and comorbidities a few months of follow-up after their first or second dose. We need a complete risk management and safety monitoring (pharmacovigilance) system, which determines the potential side effects. Similar to other vaccines, COVID-19 vaccines can cause mild or moderate side effects within a few days after injection. Some side effects such as headache, muscle pain, fatigue, fever, diarrhea, and chills have been reported, and most have happened during the first 48 h after vaccination. Therefore, subjects should continually monitor to distinguish adverse events [15].

WHO is aware that some people may show a severe allergic reaction to the vaccines (e.g., anaphylaxis). According to The United States Centers for Disease Control and Prevention (CDC) report, 11.1 per million cases of vaccinated people reported anaphylaxis in the USA [19]. If the subjects report a history of anaphylaxis with previous vaccines, they are advised not to take the new vaccine. Polyethylene glycol (PEG) and PEG derivatives (e.g., polysorbates) are probably responsible for anaphylaxis [13]. It has been recommended that before vaccination, people should notify the healthcare workers about any anaphylaxis they may have had previously. It has been proposed that all vaccinated cases remain at the vaccination site for 30 min to detect any serious side effects. It has been reported that the AstraZeneca and Johnson & Johnson/Janssen vaccines may have a possible link to a very rare side effect of unusual blood clots combined with low levels of platelet levels [7,20].

Various vaccines entered into clinical trials in a short time and were conditionally approved in less than one year. This unique speed was motivated by the timely detection of novel coronavirus genomic sequences, strong collaboration among the research centers, sufficient funding, and the urgent/huge market demand. Since the beginning of the COVID-19 pandemic, many countries are competing to develop vaccines. The development of the standard vaccine is a long process, and experiments are complete in sequential steps. However, the development of COVID-19 vaccines is being fast-tracked globally. Despite the significant progress, the safety and quality of various vaccines are the main concern. The UK, Germany, USA, and China have developed vaccines in phase 4 (post-market studies) [21].

## 7 COVID-19 vaccines dose

The Johnson & Johnson vaccine only requires one dose, while the Moderna, Pfizer-BioNTech, Oxford-AstraZeneca (in a 8–12 week interval), Sputnik V (in a 3 week interval), Novavax (in a 3 week interval), Coronavac (in a 1 month interval) need two doses. The CDC documented that while there's no priority for one vaccine over another, the vaccines aren't interchangeable.

Mixing two different vaccines can show long-lasting and strong immune responses when compared to the single vaccine. Scientists hope that mix-and-match COVID-19 vaccination regimens (e.g., e.g. AstraZeneca and Pfizer) can trigger stronger, more robust immune responses than two doses of a single vaccine. Mix-and-match COVID-19 vaccination is recognized by high levels of both T cells and antibodies, which kill infected cells and support other antiviral responses [22,23].

According to the CDC report the second dose should be injected as close to the suggested interval as possible. It may be injected up to 42 days after the first dose when a delay is inevitable. If the second dose is injected after the suitable interval, the series does not need to be restarted. Furthermore, the vaccine team should not inject second doses before the proposed interval or save or hold doses for cases who have not returned more than 42 days after their first dose [24]. The second dose of vaccine may be missed due to personal reasons or a fluctuating vaccine supply. If more than 3 weeks have passed since the first dose was received, the next dose can be injected as soon as possible [13].

## 8 COVID-19 vaccines transport and storage

Most of the available vaccines should be stored and transported in refrigeration to freezing temperatures (e.g., the Pfizer vaccine at −70 °C and Oxford-AstraZeneca 2–8 °C). Therefore, the storage and transport of mRNA vaccines is challenging. Some new vaccines can be stored at −15 to −25 °C for up to 14 days12 [25]. On the other hand, some other vaccines need ultra-cold storage (below −80 °C). That means they will be really challenging to administer effectively in poor countries or remote areas of the globe as they are far away from the central transport system. It can cause low COVID-19 immunization in these areas and, consequently, increase the endemicity of infections [25]. Care is necessary after transferring these vaccines to refrigerating to freezing temperatures or the following thawing to protect their quality. A regular schedule for temperature is vital for the preservation of stability, potency, and efficacy of COVID-19 vaccines [25]. Distribution and transportation of COVID-19 vaccines are difficult and complicated particularly in hot climate and low-income countries [26].

Stable and effective storage and transport of vaccines mean they need them at cold temperatures and transfer them quickly from the manufacturer to the medical centers. A previous report showed that 2.8 million vaccines were missed in 5 countries due to cold chain failures, and less than 10% of countries met WHO protocol for effective vaccine management [27]. Interestingly, nearly 80% of vaccine costs are related to the cold chain programme. Henceforth, the lyophilized vaccine has good stability compared with liquid form. Providing a cold chain for poor countries is the main concern. Proper preparation of lyophilized form is necessary, and powder should not be prepared until the administration. Liquid form loses its efficacy when kept at freezing temperatures because slow freezing leads to great stress to the colloids and increased aggregations [28]. Cold chain technology is needed for the liquid form, which can be challenging for use in poor countries. Appropriate cold chain infrastructure can prevent up to 25% vaccine loss in poor countries [8].

## 9 COVID-19 vaccine distribution

Many people in poor and middle-income countries may not be receiving vaccines; therefore, equitable COVID-19 vaccine distribution is essential. More than 700 million COVID-19 vaccines have been injected globally; low-income countries received only 0.2%, while wealthy countries have received more than 87%. On average, 1 in more than 500 people in poor countries has received COVID-19 vaccines, compared with 1 in 4 people in wealthy countries [13].

As of May 11, 2021, about 1.32 billion people had received the COVID-19 vaccine worldwide, equal to 17 doses for every 100 people. Some countries (e.g., Gibraltar and Israel) had vaccinated 78% of people, while Mauritius, Pakistan, Guyana, Cambodia, Albania, Bolivia, and Ecuador had less than 0.1 doses administered per 100 people. It is a disappointment that healthcare workers are dying in various countries, showing a global moral failure in these regions. Researchers believe that this uneven administration pattern can also cause virus mutations and new vaccine-resistant variants [25].

Many poor countries have low socioeconomic status (SES) with low income, high unemployment rates and poor education. These conditions may potentially influence the vaccine-accepting and purchasing processes of their people. The geographical landscape of some poor countries poses a substantial challenge to COVID-19 vaccine distribution. High altitude areas within Hindu-Kush Himalayan regions, such as Pakistan, Bhutan, Nepal, and Afghanistan, make it very difficult for health workers to distribute COVID-19 vaccines. The problematic condition may be aggravated in the desert, and remote areas participated in the war, conflict, and instability. In this respect, more than 160 million subjects have been expected to be at risk of COVID-19 vaccine inaccessibility in Syria, Yemen, Ethiopia, and South Sudan [25].

## 10 COVID-19 vaccine for children and pregnant women

COVID-19 infection has been a more dangerous and severe disease among older people. Most of the vaccines are commonly offered to adults first to avoid exposing children who are still growing and developing. Because of the high risk of severe disease in the children, elderly, immunocompromised subjects, and pregnant women, the vaccination programme should be conducted with care [10]. COVID-19 vaccine teams need to follow-up pregnancies long-term to recognize effects on infants and pregnancy.

The mRNA vaccines (Pfizer-BioNTech and Moderna) do not have the live coronavirus that leads to COVID-19 and, consequently, cannot infect. Moreover, the mRNA vaccines do not interact with an individual's DNA or lead to genetic alterations since the mRNA does not enter the cell's nucleus. The viral vector vaccines (J&J/Janssen vaccine) can be administered to pregnant women in all trimesters of pregnancy (like the Ebola vaccine). However, there are various types of COVID-19 vaccines, and our direct knowledge is currently limited about their effects during pregnancy. The efficacy and safety of COVID-19 vaccines in lactating women, the impact of COVID-19 vaccination on the breastfed infant, and effects on milk excretion or production have not been determined. However, non-replicating COVID-19 vaccines pose no risk for lactating women or their babies; hence lactating women may safely be vaccinated [29].

## 11 COVID-19 infection transmission in vaccinated people

The risks of COVID-19 in vaccinated subjects cannot be entirely eliminated as long as there is continued public transmission of the virus. Vaccinated subjects can still get COVID-19 and spread it to other people. Hence, the COVID-19 test and self-quarantine are required for travellers. Some vaccinated subjects later exposed to the coronavirus still get COVID-19. In this context, a fully vaccinated person should continue to wear a face mask, maintain social distance, and follow health care recommendations. Preliminary data from some countries showed that the viral load was 4–fold lower among those fully vaccinated with an effective vaccine. This finding suggests that viral transmission from fully vaccinated people is lower, as viral load has been recognized as the main factor for virus transmission [30]. So far, SARS-CoV-2 has not been detected in breast milk, and there are no recognized cases of transmission of virus to the infant through breast milk. However, infected women may select to breastfeed with protections to prevent transmission of the virus through respiratory droplets. Some newborns have shown COVID-19 shortly after birth. It is unknown if these newborns got the virus after, during, or before birth [31].

## 12 Low intends to take COVID-19 vaccine

It has been reported that about 15–20% of adults do not intend to take the COVID-19 vaccine. People who don't intend to get the COVID-19 vaccine are at higher risk of transmitting and contracting the virus. They can also enormously increase the pandemic period, contributing to spikes in COVID-19 cases and facilitating viral replication and the emergence of new viral variants. Common concerns among the people, who do not intend to get the COVID-19 vaccine, include the efficacy, safety, and the perceived hasty timeline for vaccine production. African American race, younger age, people with lower education, and conservative political ideology has lower intention to get COVID-19 vaccine. Receiving health care recommendations and having more fear of severe disease were both accompanied with more intention to vaccinate [11,32].

## 13 Conclusion

In conclusion, there are various types of vaccines worldwide. However, additional studies are necessary to determine the effectiveness of the COVID-19 vaccine against variants of concern. COVID-19 vaccines have obtained emergency use and there are various limitations such as vaccine distribution, variants of concern, vaccination willingness, herd immunity, vaccine efficacy, vaccine safety, and vaccine dose. To combat the current pandemic, manufacturers and healthcare authorities should work together to provide appropriate and adequate vaccinations for the prevention of COVID-19. Healthcare authorities should constantly update COVID-19-related information. Furthermore, vaccine booster doses may be required for several reasons; inadequate protection, reduced protection against new variants, and waning protection against disease or infection. However, the rationale for COVID-19 vaccine booster doses may vary by vaccine product, risk group epidemiological setting, and vaccine coverage rates.

## Funding

This research did not receive any specific grant from funding agencies in the public, commercial, or Non-Profit sectors.

## Ethics approval

Not applicable.

## Availability of data and material

Data are available upon reasonable request.

## CRediT authorship contribution statement

**Alireza Tavilani:** Conceptualization, Visualization, Data curation, Validation, Writing – original draft. **Ebrahim Abbasi:** Project administration, Visualization, Validation, Supervision, Data curation, Writing – review & editing. **Farhad Kian Ara:** Software, Writing – review & editing. **Ali Darini:** Validation, Writing – original draft, Writing – review & editing. **Zahra Asefy:** Validation, Visualization, Writing – original draft, Writing – review & editing.

## Declaration of competing interest

None to be declared.
